# Efficacy of erector spinae plane block with liposomal bupivacaine for postoperative analgesia in patients undergoing open spinal surgery: a randomized controlled trial

**DOI:** 10.3389/fphar.2026.1823099

**Published:** 2026-06-09

**Authors:** Weilian Wang, Han Xiang, Jie Gong, Xiaoqin Wu, Jinliang Xiao, Kun Zhang, Junhui Zhou

**Affiliations:** 1 Department of Anesthesiology, Jingzhou Hospital Affiliated to Yangtze University, Jingzhou, Hubei, China; 2 Department of Operating Room, Jingzhou Hospital Affiliated to Yangtze University, Jingzhou, Hubei, China; 3 Department of Pain Medicine, Jingzhou Hospital Affiliated to Yangtze University, Jingzhou, Hubei, China; 4 Department of Anesthesiology, Henan Provincial Chest Hospital, Chest Hospital of Zhengzhou University, Zhengzhou, Henan, China

**Keywords:** Liposomal bupivacaine, pain management, quality of recovery, ropivacaine, spinal surgery

## Abstract

**Background:**

Liposomal bupivacaine (LB) provides prolonged postoperative analgesia. This study aimed to evaluate the analgesic efficacy of erector spinae plane block (ESPB) using LB in patients undergoing spinal surgery.

**Methods:**

A total of 64 patients undergoing elective spinal surgery were randomly assigned in a 1:1 ratio to receive ESPB with 0.44% liposomal bupivacaine (LB group) or 0.4% ropivacaine (RH group) for postoperative pain management. The primary outcome was the incidence of rescue analgesia on the first postoperative day. Secondary outcomes included the incidence of rescue analgesia on the second and third postoperative days, visual analog scale (VAS) scores at various postoperative time points; time to first use of rescue analgesic; the cumulative area under the curve (AUC) of VAS scores, the Riker Sedation-Agitation Scale (RSAS) score at tracheal extubation; Quality of Recovery-15 (QoR-15) scores; opioid consumption; patient satisfaction and adverse events.

**Results:**

The RH group demonstrated a significantly higher incidence of rescue analgesic use than the LB group on postoperative days 1, 2, and 3 (37.5% vs. 9.4%, 59.4% vs. 18.8%, and 65.6% vs. 28.1%, respectively; all *P* < 0.05) and required their first rescue analgesic significantly earlier. VAS scores were higher in the RH group at 6, 12, 24, and 48 h postoperatively, with no significant differences observed at 1 and 72 h. Sufentanil consumption was also significantly higher in the RH group at all postoperative intervals except the first hour. Additionally, the RH group exhibited higher VAS-AUC values, lower QoR-15 scores over the first three postoperative days, and lower postoperative satisfaction scores. The incidence of nausea and dizziness was higher in the RH group, while no respiratory depression was observed in either group.

**Conclusion:**

ESPB with LB provides prolonged postoperative analgesia, reduces opioid consumption, and enhances recovery quality following spinal surgery. These findings support the incorporation of LB as a valuable component of multimodal analgesia protocols for spinal procedures.

**Clinical Trial Registration:**

https://www.chictr.org.cn/showproj.html?proj=265855, identifier ChiCTR2500100055.

## 1 Introduction

Lumbar fusion is a standard surgical procedure for various spinal disorders but often results in significant postoperative pain due to disruption of the spinal anatomy and paraspinal muscles. Although opioid-based patient-controlled intravenous analgesia (PCIA) is commonly used for postoperative pain management, its efficacy is often suboptimal and associated with adverse effects, including nausea, vomiting, abdominal distension, and pruritus (Patel et al., 2020; Podder et al., 2025). Inadequate postoperative analgesia may delay recovery, increase the risk of thromboembolism, prolong hospitalization, and raise the incidence of chronic postsurgical pain (Gan, 2017).

In 2016, Forero et al. first described the ESPB, in which local anesthetic is injected between the erector spinae muscle and the transverse process, allowing diffusion within the fascial plane to achieve analgesia (Forero et al., 2016). Previous studies have demonstrated that ESPB provides effective analgesia after spinal surgery (Kanna et al., 2022; Sun et al., 2023). However, the duration of single-shot local anesthetic injection is limited, and rebound pain, along with increased postoperative opioid consumption, remains a common concern (Chung et al., 2025; Yang et al., 2024). Continuous nerve block via catheter placement carries risks of infection, hematoma, and catheter displacement; moreover, the catheter may interfere with the surgical field and often requires specialized equipment, limiting its routine clinical use (Ilfeld, 2017; Kalimeris et al., 2020). The use of various adjuvants to prolong the duration of local anesthetics is currently supported by limited evidence and may carry risks associated with off-label use. Liposomal bupivacaine is a long-acting local anesthetic formulation that provides slow and sustained release after a single injection, thereby extending the duration of analgesia (Ilfeld et al., 2021). However, the extent to which LB prolongs analgesia remains uncertain and controversial. Nguyen et al. summarized the results of ten studies and concluded that there is insufficient evidence to support the routine use of LB for postoperative pain management following spinal surgery (Nguyen et al., 2021). In contrast, Mohammad Daher et al. suggested that LB may serve as an effective option or adjunct to enhance postoperative analgesia in patients undergoing spinal surgery (Daher et al., 2025). Therefore, additional standardized prospective studies are warranted in order to more clearly evaluate the efficacy of LB in spinal surgery.

With advances in multimodal analgesia, ultrasound-guided nerve blocks are increasingly utilized for postoperative pain management and enhanced recovery (Cozowicz et al., 2020; Maheshwari et al., 2020). Ropivacaine is often preferred for postoperative analgesia due to its lower neurotoxic and cardiotoxic profiles and less motor blockade than bupivacaine (Kuthiala and Chaudhary, 2011). However, the efficacy of long-acting LB in multimodal analgesia after spinal surgery is uncertain.

Therefore, we conducted this study to evaluate the efficacy of ESPB with LB for postoperative analgesia in patients undergoing open spinal surgery. Our primary hypothesis was that ESPB with LB reduces the need for rescue analgesia during the first postoperative day. Secondarily, we hypothesized that ESPB with LB would not only provide effective and safe analgesia but also enhance the quality of postoperative recovery following spinal surgery.

## 2 Materials and methods

### 2.1 Study design and participants

This randomized, double-blind, controlled clinical trial was conducted at Jingzhou Hospital Affiliated to Yangtze University between April 8 2025 and October 24 2025. This research aimed to evaluate the analgesic efficacy of ESPB using LB in patients undergoing spinal surgery. All patients scheduled for elective spinal surgery were randomized in a 1:1 ratio to receive ESPB with 0.44% LB (LB group) or 0.4% ropivacaine (RH group) for postoperative pain management.

Eligible patients were scheduled for elective open spinal surgery involving two or fewer vertebral segments (located at L4-L5 and/or L5-S1) under general anesthesia for the first time. Inclusion criteria were: age 18–70 years, body mass index (BMI) 18–30 kg/m^2^, and American Society of Anesthesiologists physical status (ASA-PS) I-III. Exclusion criteria included severe cardiopulmonary disease, infection at the puncture site, coagulopathy, allergy to the study drugs, intraoperative blood loss >500 mL, history of substance abuse, morbid obesity, severe hepatic or renal dysfunction, or inability to communicate effectively.

### 2.2 Randomization and blindness

All eligible patients were randomly assigned to the LB or the RH group in a 1:1 ratio. Randomization was performed using a sealed envelope method, with the random sequence generated by SPSS (version 26.0, United States). Group allocation codes, concealed in opaque envelopes were opened after the patient entered the preparation room, and study medications were prepared according to the assigned group by a nurse. ESPB was performed by the same experienced anesthesiologist who was not involved in intraoperative anesthesia management or data collection. The attending anesthesiologist was responsible for intraoperative anesthesia management. A trained and blinded research assistant assessed the patient outcomes at various time points in the post-anesthesia care unit (PACU) and the ward. Patients, clinicians, and outcome assessors remained blinded to group assignment throughout the study.

### 2.3 ESPB procedure and anesthesia management

Before surgery, all patients underwent a routine preoperative assessment in the ward and were instructed to fast for at least 8 h and abstain from water for 4 h. The study protocol, potential benefits and side effects of the ESPB technique, and the correct use of PCIA were also explained to the patients.

All ESPBs were performed before induction of general anesthesia. Patients received standard monitoring, including continuous electrocardiography, pulse oximetry, and noninvasive blood pressure measurement, with supplemental oxygen administered via a nasal cannula in the preparation room. Intravenous sufentanil 5 µg and midazolam 1 mg were given for sedation and analgesia. Patients were then positioned prone, and the skin was disinfected with iodophor. A low-frequency convex array probe (2–5 MHz, M7, Mindray Bio-Medical Electronics, Shenzhen, China) was covered with a sterile sheath and placed longitudinally in a parasagittal position (3 cm lateral to the midline) at the sacrococcygeal region. The probe was scanned up and down to locate the sacrum as a landmark and then slid upwards to identify the L5 transverse process. A 22-gauge, 80-mm needle was inserted in-plane from a cranial to caudal. After the needle passed through the erector spinae muscle and reached the surface of the L5 transverse process, 2 mL of normal saline was injected to confirm the needle tip position. Subsequently, 20 mL of the prepared local anesthetic was injected under real-time ultrasound guidance. The same procedure was repeated on the contralateral side for bilateral blockade. For the LB group, the drug was prepared by diluting 20 mL of 1.33% liposomal bupivacaine with 40 mL of normal saline to create a 0.44% mixture. For the RH group, the drug was prepared by diluting 20 mL of 1% ropivacaine with 30 mL of normal saline to create a 0.40% mixture. Thirty minutes after the block, sensory assessment was performed bilaterally in the surgical area using an ice cube by a blinded research assistant, and patients were closely monitored. Block success was defined as a reduction in sensation within the skin distribution surrounding the needle insertion site. If no sensory reduction was observed within 30 min, patients remained in their originally assigned groups according to the intention-to-treat principle.

Upon arrival in the operating room, all patients underwent standard monitoring. General anesthesia was induced with midazolam 0.05 mg/kg, propofol 2–2.5 mg/kg, sufentanil 0.5 µg/kg, and rocuronium 0.6–0.8 mg/kg. Tracheal intubation was successfully performed using a video laryngoscope. Mechanical ventilation was subsequently established, maintaining end-tidal carbon dioxide between 35–45 mmHg. An appropriate depth of anesthesia was maintained using a continuous infusion of remifentanil (0.1–0.2 µg/kg/min) and inhalation of 2 - 3 vol% sevoflurane in an oxygen-air mixture, with doses adjusted according to the patient’s condition and the surgical course. Haemodynamics during surgery were maintained within 20% of baseline. At the end of surgery, following recovery of respiration and consciousness, patients were extubated, transferred to the PACU, and subsequently returned to the ward for observation once predetermined criteria were met.

### 2.4 Postoperative analgesia protocol and rescue analgesic

Approximately 30 min before the end of surgery, all patients will receive intravenous sufentanil 10 ug and ondansetron 4 mg, followed by standardized postoperative PCIA with sufentanil. The PCIA pump was set up with sufentanil 150 µg, ondansetron 8 mg, diluted with normal saline to a total volume of 150 mL, without background dose. The pump was administered when requested by the patient with a single dose of 2 mL and a lockout interval of 20 min, with a limit of 8 mL/4 h. Parecoxib sodium 20–40 mg was given as rescue analgesia when PCIA was not sufficient to relieve postoperative pain and the patient requested additional analgesics.

### 2.5 Outcome measurements

Outcome measurements were collected by an investigator trained in standardized assessment protocols, who was blinded to participant group allocation. Patient follow-up assessments were conducted from 1 day preoperatively to 3 days postoperatively to evaluate the benefits and potential adverse effects of the intervention.

Baseline data were recorded for all patients, including patient demographics. ASA classification was recorded the day before surgery. Intraoperative data, including the duration of surgery, extubation time, estimated blood loss, and the number of surgical segments, were collected on the day of surgery.

The primary outcome was the incidence of rescue analgesia on the first postoperative day. Rescue analgesia was defined as the additional use of parecoxib sodium for postoperative analgesia.

Secondary outcomes included the time to first use of rescue analgesic in the ward (defined as the time from the end of surgery to the patient’s first request for rescue analgesia); the incidence of rescue analgesia on the second and third postoperative days; Pain intensity was assessed using the VAS at 1, 6, 12, 24, 48, and 72 h postoperatively. The VAS score ranges from 0 (no pain) to 10 (worst imaginable pain). Other outcomes included the VAS-AUC, sufentanil consumption, and the RSAS score at tracheal extubation (1 = Unarousable, 2 = Very sedated, 3 = Sedated, 4 = Calm, cooperative, 5 = Agitated, 6 = Very agitated, 7 = Dangerous agitation) (Riker et al., 1999). Recovery quality was assessed 1 day before surgery and at 24, 48, and 72 h postoperatively using the QoR-15 scale. This scale consists of 15 items, each scored from 0 to 10, evaluating pain, physical comfort, physical independence, psychological state, and emotional state. The total score ranges from 0 to 150, with higher scores indicating better recovery quality. Kaplan-Meier survival analysis was performed to evaluate postoperative opioid-free time in both groups, with comparisons conducted using the log-rank test. Patient satisfaction with pain control was assessed at 72 h postoperatively using a 5-point Likert scale (0 = extremely dissatisfied to 4 = extremely satisfied).

Adverse events were recorded from the initiation of anesthesia to 72 h postoperatively. These included respiratory depression, defined as a spontaneous respiratory rate below 8 breaths/min or oxygen saturation below 90%; postoperative nausea and vomiting (PONV); dizziness and drowsiness.

### 2.6 Statistical analysis and sample size

Statistical analyses were conducted using IBM SPSS (version 26.0, USA) and GraphPad Prism (version 9.2.0, USA). Levene’s test was used to assess the homogeneity of variances, and the Shapiro-Wilk test was used to check for normality. Normally distributed continuous data were presented as means ± standard deviation (SD) and compared using two-sample t-test. Non-normally distributed data were presented as median (interquartile range [IQR]) and compared using Mann-Whitney *U* test. Kaplan-Meier survival analysis was performed to determine the postoperative opioid-free times in both groups, with comparisons conducted using the log-rank test, hazard ratio (HR) and 95% CI calculated using univariate Cox regression analysis. Categorical variables were presented as frequencies (%) and compared using the Pearson’s chi-squared test or Fisher’s exact test. All statistical tests were two-sided, and *P* < 0.05 was considered statistically significant. All data were analyzed according to the intention-to-treat principle, with missing values addressed using multiple imputation.

Sample size calculation was performed using Power Analysis and Sample Size software (version 15.0, USA). In our preliminary study, the incidence of rescue analgesics at 24 h after surgery was 47% in the RH group and 10% in the LB group. With a significance level of α = 0.05 and power (1-β) = 0.80, analysis indicated that 28 participants were needed per group. To account for an 10% dropout rate, a total of 64 patients were planned to be included, with 32 patients in each group.

## 3 Results

### 3.1 Study flow and baseline characteristics

**FIGURE 1:**
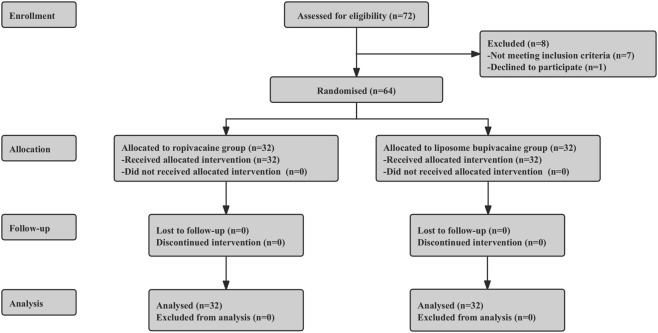
Consolidated Standards of Reporting Trials (CONSORT) diagram of patient flow in this study.

A total of 72 patients were screened during the study from April 8 2025 to October 24 2025. Eight patients were excluded due to failure to meet the inclusion criteria or declined to participate before randomization. The remaining 64 patients were randomly assigned to RH or LB groups in a 1:1 ratio (32 patients per group), and all these patients successfully completed the study and were included in the final analysis. The Consolidated Standards of Reporting Trials (CONSORT) flow diagram was shown in Figure 1. The supplementary material is cited in the following sentence: CONSORT checklist can be found in Supplementary Table 1. Baseline characteristics and surgical conditions showed no significant differences between the two groups (Table 1).

**TABLE 1:** Baseline characteristics and intra-operative data.

Variables	RH group (n = 32)	LB group (n = 32)	t/χ^2^ value	*P*-value
Sex; male	17 (53%)	18 (56%)	0.063	0.802
Age; years	58.7 ± 8.6	60.9 ± 6.6	−1.160	0.250
Weight; kg	65.63 ± 12.28	64.75 ± 10.14	0.757	0.757
BMI; kg/m^2^	24.5 ± 3.9	23.9 ± 3.2	0.662	0.510
ASA physical status				
2	19 (59%)	15 (47%)	1.004	0.316
3	13 (41%)	17 (53%)		
Duration of surgery; min	191.5 ± 32.8	201.1 ± 37.3	−1.094	0.278
Tracheal extubation time; min	13.7 ± 1.9	14.4 ± 1.6	−1.554	0.125
Number of fused levels				
1	20 (63%)	16 (50%)	1.016	0.313
2	12 (37%)	16 (50%)		
Fluid infusion volume; ml	1,448 ± 403	1,412 ± 447	0.338	0.737
Blood loss volume; ml	242 ± 76	257 ± 71	−0.849	0.399
RSAS	4 (4, 4)	4 (4, 4)	−1.675	0.094

Values are presented as mean ± SD, number (proportion) or median (IQR [range]).

Abbreviations: RH, ropivacaine group; LB, liposomal bupivacaine group; RSAS, riker sedation agitation scale.

### 3.2 Primary outcomes

**FIGURE 2:**
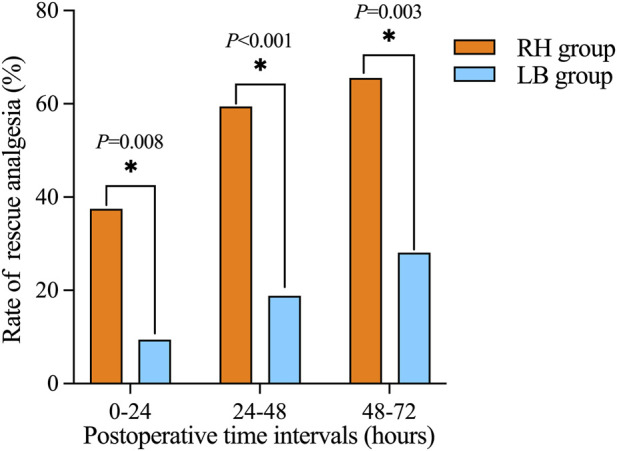
Rate of rescue analgesia. Notes: Values are presented as proportion. *P < 0.05 was considered statistically significant. Abbreviations: RH, ropivacaine group; LB, liposomal bupivacaine group.

The incidence of rescue analgesic use was significantly higher in the RH group compared to the LB group on the first postoperative days (POD1: 37.5% vs. 9.4%, *P* = 0.008) (Figure 2).

### 3.3 Secondary outcomes

#### 3.3.1 Rescue analgesic after surgery

**FIGURE 3:**
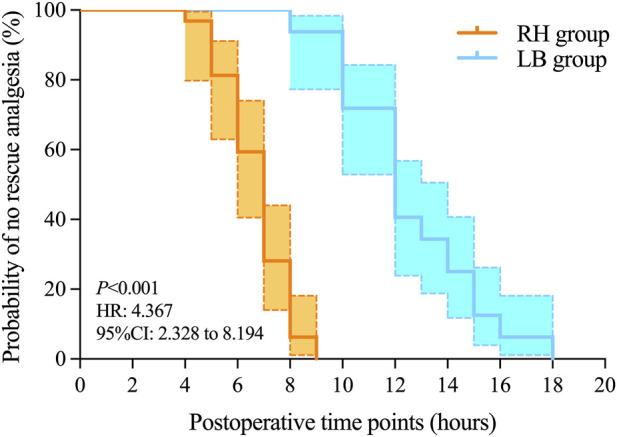
Survival curve of time to first rescue. Notes: Solid line represents the trajectory of median values, while shaded area denotes 95% confidence intervals. Abbreviations: RH, ropivacaine group; LB, liposomal bupivacaine group; HR, hazard ratio; 95% CI, 95% confidence interval.

The incidence of rescue analgesic use was significantly higher in the RH group than in the LB group on the second and third postoperative days (POD2: 59.4% vs. 18.8%, *P* < 0.001; POD3: 65.6% vs. 28.1%, *P* = 0.003) (Figure 2). Furthermore, patients in the RH group required their first dose of rescue analgesics significantly earlier than those in the LB group (6.72 ± 1.25 h vs. 12.56 ± 2.56 h; HR: 4.367, 95% CI: 2.328–8.194, *P* < 0.001) (Figure 3).

#### 3.3.2 Pain score and sufentanil consumption after surgery

**FIGURE 4:**
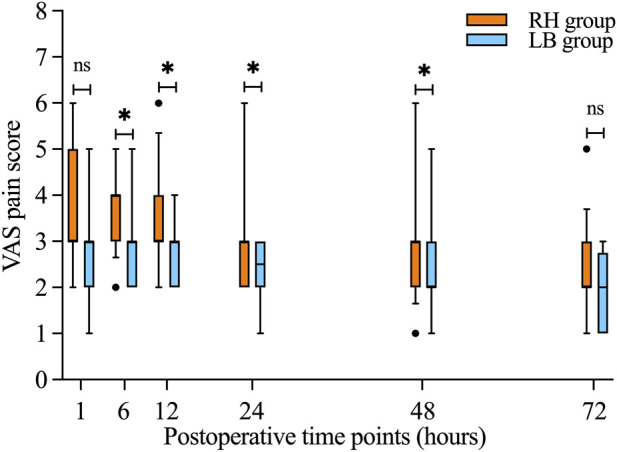
Pain intensity after surgery. Notes: Pain intensity after surgery was measured using the VAS scale. Box plots depict the median (middle line), interquartile range (box), and 5%–95% value (whiskers) for the two groups; ^*^ *P* < 0.05 was considered statistically significant. Abbreviations: RH, ropivacaine group; LB, liposomal bupivacaine group; VAS, visual analogue scale.

Pain scores showed no significant differences between the two groups at 1 h (median [IQR]: 3 [2, 3], vs. 3 [2, 3], *P* = 0.105) and 72 h (median [IQR]: 2 [2, 3], vs. 2 [1, 2.75], *P* = 0.077) postoperatively. However, the RH group exhibited higher pain scores than the LB group at 6 h (median [IQR]: 4 [3, 4], vs. 3 [2, 3], *P* < 0.001), 12 h (median [IQR]: 3 [3, 4], vs. 3 [2, 3], *P* < 0.001), 24 h (median [IQR]: 3 [2, 3], vs. 2.5 [2, 3], *P* < 0.001), 48 h (median [IQR]: 3 [2, 3], vs. 2 [2, 3], *P* < 0.001) after surgery (Figure 4).

**FIGURE 5:**
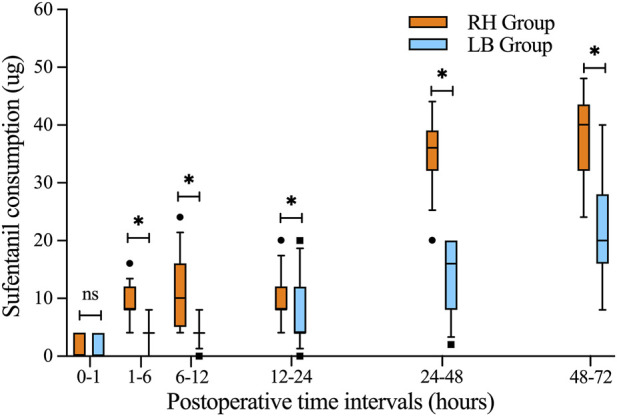
Sufentanil consumption after surgery. Notes: Sufentanil consumption after surgery was recorded using an electronic patient-controlled analgesia pump. Box plots represent the median (middle line), interquartile range (box), and 5%–95% value (whiskers) for the two groups; ^*^ *P* < 0.05 was considered statistically significant. Abbreviations: RH, ropivacaine group; LB, liposomal bupivacaine group.

There was no significant difference in sufentanil consumption between the RH and LB groups during the 0–1 h postoperative interval (median [IQR]: 0 [0, 4] vs. 0 [0, 4], *P* = 0.438). However, sufentanil consumption was significantly higher in the RH group than in the LB group at all subsequent postoperative intervals: 1–6 h (median [IQR]: 8 [8, 12] vs. 4 [4, 4], *P* < 0.001), 6–12 h (median [IQR]: 10 [5, 16] vs. 4 [4, 4], *P* < 0.001), 12–24 h (median [IQR]: 8 [8, 12] vs. 4 [4, 12], *P* = 0.025), 24–48 h (median [IQR]: 36 [32, 39] vs. 16 [8, 20], *P* < 0.001), 48–72 h (median [IQR]: 40 [32, 44] vs. 20 [16, 28], *P* < 0.001) (Figure 5).

**FIGURE 6:**
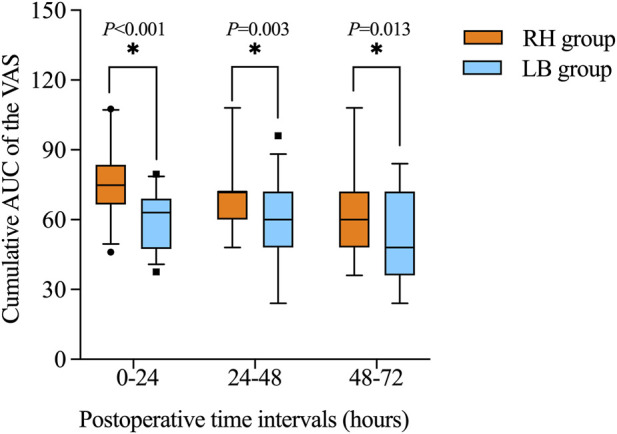
Cumulative AUC of the VAS within 72 h postoperatively. Notes: Comparison of the cumulative AUC of VAS scores within the first 72 h postoperatively between the two groups. Box plots depict median (middle line), interquartile range (box), and 5%–95% value (whiskers); ^*^ *P* < 0.05 was considered statistically significant. Abbreviations: AUC, area under the curve; RH, ropivacaine group; LB, liposomal bupivacaine group; VAS, visual analogue scale.

For the cumulative AUC of the VAS score, the RH group had higher scores than the LB group on all three postoperative days: VAS-AUC 0–24 h (median [IQR]: 75 [67, 84] vs. 63 [47, 69], *P* < 0.001), VAS-AUC 24–48 h (median [IQR]: 72 [60, 72] vs. 60 [48, 72], *P* = 0.003), VAS-AUC 48–72 h (median [IQR]: 60 [48, 72] vs. 48 [36, 72], *P* = 0.013) (Figure 6).

#### 3.3.3 Recovery quality after surgery

**FIGURE 7:**
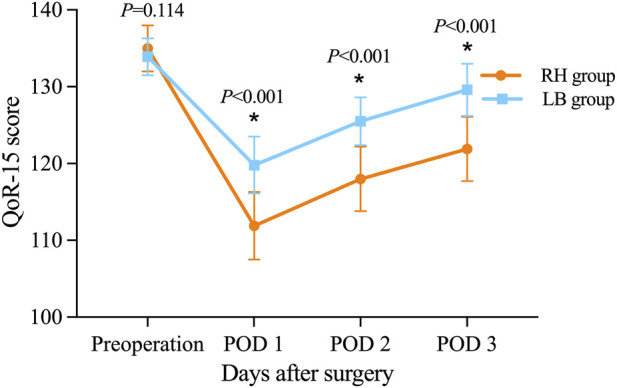
QoR-15 preoperative and postoperative. Notes: Comparison of preoperative and postoperative QoR-15 scores between the two groups; Data points represent means with standard deviation error bars. ^*^ *P* < 0.05 was considered statistically significant. Abbreviations: QoR-15, Quality of Recovery-15; RH, ropivacaine group; LB, liposomal bupivacaine group; POD, postoperative day.

There was no significant difference in the preoperative recovery quality scores between the two groups, (135.0 ± 3.0 vs. 133.9 ± 2.4, *P* = 0.114). However, the QoR-15 scores on the first, second and third postoperative days were significantly lower in the RH group compared to the LB group, (111.9 ± 4.4 vs. 119.8 ± 3.7, *P* < 0.001); (118.0 ± 4.2 vs. 125.5 ± 3.1, *P* < 0.001); (121.9 ± 4.2 vs. 129.6 ± 3.4, *P* < 0.001) (Figure 7).

#### 3.3.4 Adverse events

There were no significant differences between the RH and LB groups in the incidence of vomiting, respiratory depression, or drowsiness. However, the RH group had a significantly higher incidence of nausea compared to the LB group (40.6% vs. 12.5%, *P* = 0.011), as well as a higher incidence of dizziness (28.1% vs. 6.3%, *P* = 0.020). Additionally, postoperative satisfaction scores were lower in the RH group than in the LB group (median [IQR]: 2 [1, 3] vs. 3 [3, 4], *P* < 0.001) (Table 2).

**TABLE 2:** Adverse events and safety outcomes.

Variable	RH group (n = 32)	LB group (n = 32)	χ^2^/Z value	*P*-value
Nausea	13 (40.6%)	4 (12.5%)	6.488	0.011
Vomiting	4 (12.5%)	2 (6.3%)	0.184	0.668
Respiratory depression	2 (6.3%)	1 (3.1%)	0	1.000
Dizziness	9 (28.1%)	2 (6.3%)	5.379	0.020
Drowsiness	7 (21.9%)	3 (9.4%)	1.896	0.168
Patient satisfaction (5-point likert scale)	2 (1, 3)	3 (3, 4)	−3.707	<0.001

Values are presented as number (proportion) or median (IQR [range]).

Abbreviations: RH, ropivacaine group; LB, liposomal bupivacaine group.

## 4 Discussion

In this randomized controlled trial, we compared the efficacy of LB and ropivacaine for postoperative analgesia and recovery quality in patients undergoing spinal surgery. The results demonstrated that LB significantly reduced the proportion of patients requiring rescue analgesia within 72 h postoperatively, prolonged the time to first rescue analgesia, decreased postoperative opioid consumption, and improved recovery quality.

Anatomical studies have found that the spread of dye or local anesthetic in ESPB predominantly occurs dorsally, with minimal ventral diffusion (Sorenstua et al., 2023; Varela et al., 2024). Meta-analyses of ESPB for abdominal and thoracic procedures have also shown no significant reduction in postoperative pain scores or opioid consumption (Bhushan et al., 2022; Zhang et al., 2025). This may be attributed to the neural distribution within the erector spinae plane, as both the vertebral column and paraspinal muscles are primarily innervated by the dorsal rami. The predominant dorsal spread of anesthetic provides a theoretical rationale for the application of ESPB in spinal surgery. However, the required volume of local anesthetic varies depending on the anatomical level of the ESPB. Previous study has shown the median volume required to block a single segment is 5 mL in the lumbar region (De Cassai et al., 2020). Considering the spread of local anesthetic and the maximum safe dosage for a single administration, this study included only patients undergoing procedures involving two or fewer spinal segments, ensuring the local anesthetic could adequately cover the surgical area while maintaining safety. Interestingly, ESPB in the thoracic region typically requires 3.3–3.5 mL to cover one vertebral, therefore, the spread of even small volumes of local anesthetic in thoracic ESPB, will lead to multilevel analgesia (De Cassai et al., 2020). This may be due to the different anatomy of vertebrae and the different spinal curves. As a result, it should be kept in mind that ESPB from different levels may lead to different local anesthetic spread and therefore varying anesthetic effects (Kose et al., 2018). Evidence suggests that ultrasound-guided bilateral ESPB provides effective analgesia after open lumbar surgery, reducing opioid consumption and pain scores without serious complications (Lin et al., 2022; Wittayapairoj et al., 2023; Zhang et al., 2021). However, previous studies have primarily utilized ropivacaine, achieving significant reductions in pain scores and opioid requirements only during the first 10 h postoperatively, with no differences between groups thereafter. These findings are consistent with our results, in which the first rescue analgesia in the ropivacaine group was required at approximately 8 h postoperatively.

Effective control of postoperative pain is important for improving recovery quality and preventing emergence agitation (Wang et al., 2023). In this study, no significant difference in the RSAS score at recovery was observed between the two groups, which may be attributed to the effective pain relief provided by the ESPB and PCIA administered after the operation. Surgical patients typically require substantial opioid administration within the first 24 h postoperatively due to severe incisional pain, which increases the risk of associated complications and delays recovery (Small and Laycock, 2020). Conventional local anesthetics provide only short-term analgesia, and the incidence of rebound pain after nerve block is as high as 49.6%–61.7%, necessitating additional opioid use for rescue analgesia (Admassie et al., 2022; Barry et al., 2021). LB is an extended-release formulation of bupivacaine approved by the U.S. Food and Drug Administration in 2011 for surgical site infiltration. The slow degradation of liposomes maintains stable tissue concentrations of bupivacaine over an extended period.

Previous studies have demonstrated that LB, when applied for paravertebral or intercostal nerve blocks, can significantly reduce postoperative opioid consumption during the first 72 h, decrease pain scores within the first 24 h, and enhance postoperative recovery quality (Shan et al., 2025; Wang et al., 2025; Ye et al., 2025). In our research, the LB group showed a significantly lower rate of rescue analgesia and a longer time to first rescue analgesic use. Pain scores at all postoperative time points, except at 1 h and 72 h, were lower in the LB group, which may primarily reflect differences in the duration of action and pharmacokinetic profiles between the two drugs rather than a specific advantage of ESPB itself. All ESPB procedures were performed preoperatively, and the average duration of surgery was approximately 3 h, the analgesic effect of ropivacaine lasted 8–10 h, whereas LB provided analgesia for approximately 72 h. Therefore, both groups were within the effective analgesic timeframe at the first hour after surgery, and the analgesic effects had largely dissipated in both groups at 72 h postoperatively, resulting in no significant differences in pain scores at these time points. These findings are consistent with previous research indicating that LB reduces postoperative opioid use and pain scores (Dincer et al., 2023; Niu et al., 2025). Additionally, this study assessed the overall postoperative analgesic effect by observing the AUC of the VAS from 0 to 72 h, thereby avoiding biases from relying on a single time point analysis. The results showed that cumulative pain scores over 3 days were consistently lower in the LB group. Since LB costs about 100 times as much as conventional anesthetic. Even if a reduction in VAS is clinically meaningful for some patients, it is worth pondering the cost-benefit balance of whether the substantial increase in costs associated with routine use of LB for postoperative analgesia is justified, especially in resource-constrained healthcare systems.

Current guidelines clearly recommend the use of local anesthetic techniques or fascial plane blocks in spinal surgery to optimize early mobilization and discharge outcomes (Debono et al., 2021). Multimodal analgesia strategies based on peripheral nerve blocks are gradually replacing opioid-based regimens (Yin et al., 2025). Furthermore, the 2026 Practice Guidelines for Acute Pain Management in the Perioperative Setting, released by the ASA emphasize the importance of local and regional analgesia as key components of multimodal pain management, highlighting their role in relieving postoperative pain and reducing opioid consumption (Joshi et al., 2026). The analgesic efficacy of all fascial plane blocks depends on the spread of local anesthetic within the tissue plane, which may vary among individuals and is influenced by multiple factors (Elsharkawy et al., 2018). In our research, all patients received a multimodal postoperative pain management protocol consisting of ESPB combined with intravenous analgesia, and no serious adverse events occurred in either group. Sufentanil consumption was higher at all postoperative intervals postoperatively in ropivacaine group, which was accompanied by a higher incidence of nausea and dizziness and lower patient satisfaction scores. These findings may be attributed to the more sustained analgesic effect of LB, resulting in more effective pain control over an extended period, reduced opioid requirements, and an overall improved recovery experience during hospitalization which is consistent with previous reports (Cai et al., 2020). Although these regimens in our research are commonly used in clinical practice, true pharmacologic equivalence is uncertain (Bergese et al., 2012; Hu et al., 2013). Differences in potency, duration of action, and tissue distribution may have contributed to the observed differences in analgesic outcomes. Future studies should systematically investigate the optimal concentration and volume of both agents to ensure more meaningful comparison.

Postoperative recovery quality was evaluated using the QoR-15 score in our research, which encompasses multiple dimensions of recovery, including pain, psychological state, emotional state, physical independence, and comfort. Compared with single physiological indicators, QoR-15 provides a more comprehensive assessment of patient recovery and more accurately reflects the overall impact of surgery on postoperative outcomes. Previous studies have shown that continuous ESPB with ropivacaine achieves analgesic effects comparable to intravenous analgesia, while ESPB can further improve postoperative recovery quality, particularly by enhancing sleep quality and reducing anxiety (Hu et al., 2025). Similarly, in our study, the LB group demonstrated significantly better postoperative recovery quality, suggesting that effective pain control contributes to improved postoperative recovery.

Despite these findings, this study has several limitations. First, due to the different pharmacokinetic and duration of action of the two drugs, the results may reflect the superiority of drug formulation rather than efficacy of ESPB in patients undergoing spinal surgery. Second, as a single-center study with a limited sample size, the findings may not be broadly applicable. Therefore, multicenter studies with larger cohorts are needed to enhance the generalizability of the results and may provide additional clinically relevant insights. Third, we did not systematically investigate varying concentrations and volumes of local anesthetics, but instead focused on a commonly used dose. Whether alternative concentrations or volumes could optimize analgesic outcomes remains uncertain. In clinical situations involving extreme conditions or highly vascularized areas, there is a risk of local anesthetic systemic toxicity (LAST) due to potential overdose, and cases of LAST associated with LB have been reported (Aggarwal, 2018). Consequently, researchers are now exploring the feasibility of sustained-release formulations of ropivacaine, which may represent a future direction for pain management (Pope et al., 2025). Finally, the study focuses on acute postoperative recovery up to 72 h. Since postoperative pain may contribute to persistent pain syndromes and prolonged opioid use. Chronic postsurgical pain is closely associated with psychological disorders such as anxiety and depression, whether effective postoperative pain control leads to improved long-term outcomes require further investigation.

## 5 Conclusion

In conclusion, our study demonstrated that ESPB with LB provides prolonged postoperative analgesia, reduces opioid consumption, and improves recovery quality following spinal surgery. These findings support the use of LB as a valuable component of multimodal analgesia protocols for spinal procedures. Future studies should focus on optimizing application strategies and evaluating the long-term benefits of this approach.

## Data Availability

The original contributions presented in the study are included in the article/Supplementary Material, further inquiries can be directed to the corresponding author.
